# Anthropometric Obesity Phenotypes in Young Physically Active Men: The Role of Body Composition and Fat Distribution

**DOI:** 10.3390/life15121808

**Published:** 2025-11-25

**Authors:** Agnieszka Wasiluk, Jerzy Saczuk, Ryszard Asienkiewicz

**Affiliations:** 1 Department of Health Promotion, Faculty of Physical Education and Health in Biala, Podlaska, Jozef Pilsudski University of Physical Education in Warsaw, 00-968 Warsaw, Poland; 2 Institute of Sport, Tourism and Nutrition, Faculty of Science and Natural Sciences, University of Zielona Góra, 65-417 Zielona Góra, Poland

**Keywords:** anthropometric obesity, body composition, lean body mass, fat distribution, young adults, physically active

## Abstract

Background: BMI does not distinguish fat from lean mass, reducing its usefulness in active young adults. This study assessed obesity phenotypes and their associations with lean mass and adipose tissue distribution. Methods: A total of 174 male university students (mean age 20.88 ± 0.86 years) underwent anthropometric measurements, including BMI, waist circumference (WC), and waist-to-hip ratio (WHR), and body composition assessment using bioelectrical impedance analysis (BIA). Participants were classified into four phenotypes (MHNW, MHO, MUO, and NWO) based on BMI, body fat percentage (%BF), WC, and WHR. One-way ANOVA with Newman–Keuls post hoc tests was used to assess group differences. A multiple linear regression model was applied to determine the independent predictors of %BF. Results: MHNW was the most prevalent phenotype (66.7%), followed by MHO (19.0%), MUO (8.0%), and NWO (6.9%). Significant differences were observed in body composition and fat distribution among phenotypes. BMI poorly reflected %BF. Multiple regression analysis identified WC and WHR as the strongest independent predictors of %BF, whereas BMI was not significant after adjustment. Higher lean body mass in MHNW and MHO was associated with more favorable fat distribution patterns. Conclusions: BMI alone is insufficient to assess metabolic risk in young, physically active adults. WC and WHR showed superior predictive value for body fatness and should complement BMI in metabolic risk evaluation. Including lean body mass and fat distribution measures improves the identification of individuals at risk of metabolic and cardiovascular disorders.

## 1. Introduction

Obesity is one of the most urgent public health issues of the 21st century, linked to higher rates of cardiovascular diseases, type 2 diabetes, some cancers, and lower quality of life [1,2]. Worldwide, the rates of overweight and obesity are increasing rapidly among both adults and teenagers [3,4]. Therefore, early detection of individuals at risk for negative metabolic effects of excess weight has become a vital goal. In clinical practice and epidemiological research, the body mass index (BMI) remains the most widely used indicator for assessing overweight and obesity, primarily due to its simplicity and broad applicability [5]. Beyond anthropometric indicators, lifestyle factors such as diet and structured physical activity play a key role in mitigating the health consequences of obesity and improving quality of life. Recent findings have highlighted the benefits of integrative neuromuscular training—combining strength, coordination, and endurance exercises—in enhancing metabolic health and supporting weight management in individuals with obesity [6].

However, despite its widespread acceptance, BMI has considerable limitations that may compromise the accuracy of nutritional status assessment. It is based solely on body weight and height, without distinguishing between fat mass and lean mass, which can lead to misclassification of metabolic health [7]. These limitations are particularly relevant in young, physically active adults, where elevated body weight often reflects increased muscle mass rather than excess adiposity [8]. Furthermore, BMI does not account for parameters reflecting fat distribution, such as waist circumference or the waist-to-hip ratio (WHR), which are stronger predictors of metabolic risk [9]. In response to these shortcomings, researchers have increasingly sought alternative indicators that more accurately capture the health risks associated with obesity [10].

To better understand the anthropometric heterogeneity of individuals with similar BMI, the concept of obesity-related anthropometric phenotypes has been introduced. The most commonly described include: normal-weight obesity (NWO), characterized by normal BMI but elevated body fat percentage; “metabolically healthy obesity (MHO)” and “metabolically unhealthy obesity (MUO),” terms retained for consistency with previous literature but referring in this study exclusively to anthropometric profiles rather than true metabolic status; and metabolically unhealthy normal-weight (MHNW) individuals, who often remain undetected during routine screenings [8,11]. To date, most studies on anthropometric obesity phenotypes have focused on overweight adults or patients with established metabolic disorders [12]. Therefore, there remains a paucity of data regarding young, physically active adults, who may exhibit a distinct body composition profile characterized by high lean mass and relatively low to moderate fat mass. Investigating these anthropometric phenotypes in this population is of particular importance, as it enables early identification of individuals who may be at risk of health-related disturbances despite an ostensibly favorable BMI. The present study aimed to examine the prevalence of obesity-related anthropometric phenotypes in a cohort of young, physically active men and to explore their associations with body composition, with particular emphasis on lean mass and anthropometric indicators of fat distribution.

## 2. Materials and Methods

### 2.1. Participants

The study included 174 male undergraduate students of physical education at the University of Biala Podlaska (Poland). All participants were adults (≥18 years old), in good general health, and regularly attended all practical and theoretical physical education classes included in the study curriculum. The sample size was determined by the total number of eligible students who met these criteria during the recruitment period. Participants with any known metabolic, cardiovascular, or endocrine disorder, or those undergoing pharmacological treatment that could affect body composition, were excluded from the study. All participants provided written informed consent before enrollment. The mean chronological age of participants was 20.88 ± 0.86 years. The study was conducted in accordance with the Declaration of Helsinki, and the protocol was approved by the local institutional ethics committee (approval no. SKE.0030.62.2025, approval date: 27 June 2025).

### 2.2. Anthropometric Measurements

Body height (cm) and body mass (kg) were measured using a calibrated stadiometer and electronic scale, with participants barefoot and wearing light clothing, in accordance with standard anthropometric protocols [13]. Body mass index (BMI, kg/m^2^) was calculated as weight divided by height squared. Waist circumference (WC, cm) and hip circumference (HC, cm) were assessed with a flexible anthropometric tape according to the recommendations of the World Health Organization [14]. The waist-to-hip ratio (WHR) was subsequently calculated, and the waist-to-height ratio (WHtR) was derived from waist circumference and height measurements.

### 2.3. Body Composition Analysis

Body composition was assessed using bioelectrical impedance analysis (BIA; Jawon Medical IOI-353, Seoul, South Korea). The following parameters were obtained: body fat percentage (%BF), fat mass (FM, kg), and lean body mass (LBM, kg). The percentage of fat-free mass (%FFM) was calculated as 100% − %BF. Fat mass index (FMI, kg/m^2^) was derived as FM divided by height squared (m^2^), and fat-free mass index (FFMI, kg/m^2^) as LBM divided by height squared (m^2^). Measurements were performed in the morning, after an overnight fast of approximately 10–12 h, following voiding of the bladder and at least 24 h after the last vigorous physical activity. Participants were instructed to refrain from caffeine and alcohol consumption for 48 h before testing. All assessments were conducted under standardized laboratory conditions to minimize potential sources of variability [15,16]. The Jawon IOI-353 analyzer uses a multifrequency (5–500 kHz) tetrapolar system and manufacturer’s proprietary equations validated in adult populations [17]. Raw impedance parameters (resistance, reactance, and phase angle) are internally measured by the device but were not accessible to the researchers, as the IOI-353 does not provide these values in its output report.

### 2.4. Phenotypic Classification

Phenotypic classification was based exclusively on anthropometric parameters (BMI, %BF, WC, WHR, and WHtR), without the inclusion of biochemical markers. Accordingly, the phenotypic labels used in this study (MHNW, NWO, MHO, MUO) denote anthropometrically defined body composition profiles rather than metabolic health categories. This approach enabled the identification of differences in body composition and fat distribution independent of laboratory measurements. Although these abbreviations originate from traditional metabolic phenotype nomenclature, they are retained here solely to maintain comparability with previous studies that applied analogous anthropometric definitions. Considering established reference values and characteristics of the studied population, the following cut-off thresholds were applied for men. Waist circumference (WC) ≥ 94 cm and waist-to-hip ratio (WHR) > 0.90 were adopted according to World Health Organization (WHO) recommendations for European populations [14]. The waist-to-height ratio (WHtR) threshold was set at 0.50, based on the criterion proposed by Ashwell and Hsieh [18]. A body fat percentage (%BF) > 20% was considered excessive adiposity, whereas values ≤ 20% were regarded as normal for young adult males [19,20]. Phenotypic classification criteria:

MHNW (Metabolically Healthy Normal Weight; anthropometrically favorable profile): BMI < 25 kg/m^2^, %BF ≤ 20%, WC < 94 cm, WHR ≤ 0.90, WHtR < 0.50.

NWO (Normal Weight Obesity Phenotype): BMI < 25 kg/m^2^, %BF > 20% and/or unfavorable fat distribution indices (WC ≥ 94 cm, WHR > 0.90, WHtR ≥ 0.50).

MHO (Metabolically Healthy Obesity; anthropometrically favorable profile): BMI ≥ 25 kg/m^2^, %BF ≤ 20%, WC < 94 cm, WHR ≤ 0.90, WHtR < 0.50.

MUO (Metabolically Unhealthy Obesity; anthropometrically unfavorable profile): BMI ≥ 25 kg/m^2^, %BF > 20% and/or unfavorable fat distribution indices (WC ≥ 94 cm, WHR > 0.90, WHtR ≥ 0.50).

### 2.5. Statistical Analysis

All variables are presented as means ± standard deviation (SD). The Shapiro–Wilk test was used to verify the normality of data distribution. One-way analysis of variance (ANOVA) was applied to assess differences in continuous variables across phenotypic groups. Post hoc Newman–Keuls tests were employed for pairwise comparisons. For increased robustness of the between-group comparisons, the outcomes of the Newman–Keuls procedure were additionally verified using more conservative post hoc approaches (e.g., Tukey HSD), which produced consistent conclusions; however, only the Newman–Keuls results are reported in the manuscript. To account for unequal subgroup sizes, the assumptions of the ANOVA procedure (normality and homogeneity of variances) were checked prior to analysis. Linear regression analyses were conducted to illustrate the associations between selected variables, with scatterplots generated for %BF versus BMI and for LBM versus WC, including the corresponding regression equations. To examine the independent contribution of anthropometric indices to body fat percentage, a multiple linear regression model was performed. Body fat percentage (%BF) served as the dependent variable, while BMI, waist circumference (WC), waist-to-hip ratio (WHR), and waist-to-height ratio (WHtR) were included as predictors. This analysis enabled the assessment of multidimensional relationships between anthropometric characteristics and indicators of adiposity. The significance level was set at *p* < 0.05. Statistical procedures were performed using Statistica 13.0 software (StatSoft, Kraków, Poland).

## 3. Results

Table 1 presents the descriptive characteristics of the study participants. The group of physically active young men demonstrated relatively homogeneous height and body mass but showed considerable interindividual variability in adiposity indices and body composition. Body mass index (BMI) values ranged from 18.59 to 35.27 kg/m^2^, covering individuals from underweight to the threshold of obesity. Despite a mean BMI within the normal range, the wide dispersion of body fat percentage (%BF, 7.23–24.01%) and fat mass index (FMI, 1.60–7.52 kg/m^2^) highlights the limitations of BMI as the sole diagnostic criterion for obesity in this population.

Participants exhibited a high proportion of fat-free mass (%FFM = 83.21%), consistent with their physical education training background. However, the variation in fat-free mass index (FFMI, 15.78–30.45 kg/m^2^) indicates substantial individual differences in muscular development. Indices of central fat distribution, including the waist-to-hip ratio (WHR) and waist-to-height ratio (WHtR), also displayed marked variability, indicating potential differences in metabolic risk profiles within the cohort.

The present observations support the use of a multiparametric classification approach to distinguish obesity phenotypes, integrating BMI, %BF, and waist-related indices to capture both total and regional adiposity. The observed heterogeneity provided the basis for further analyses aimed at identifying distinct anthropometric profiles despite comparable levels of physical activity. Within the study cohort of 174 men, the majority of participants were classified as normal weight with a favorable anthropometric profile (MHNW; n = 116, 66.7%), while the remaining 33.9% were distributed across obesity with a favorable anthropometric profile (MHO; n = 33, 19.0%), obesity with an unfavorable anthropometric profile (MUO; n = 14, 8.0%), and normal weight obesity (NWO; n = 12, 6.9%) (Table 2).

Marked differences in anthropometric and body composition parameters were observed across phenotypes. Body mass and BMI were significantly higher in the MHO and MUO groups compared with MHNW and NWO (*p* < 0.001), while no significant differences were found between MHNW and NWO. Waist circumference was elevated in all obese phenotypes and also in NWO compared with MHNW (*p* < 0.001), whereas hip circumference was greatest in MHO and MUO. Consequently, WHR and WHtR were markedly higher in NWO and MUO compared with MHNW and MHO, reflecting unfavorable fat distribution.

With regard to body composition, %BF was moderately higher in NWO, MHO, and MUO compared with MHNW, with significant differences primarily between MHNW and MUO. Fat mass (FM) and fat mass index (FMI) were consistently greater in MHO and MUO (*p* < 0.001), while lean body mass (LBM) and fat-free mass index (FFMI) were also significantly higher in these groups, distinguishing them from MHNW and NWO, which exhibited comparable values. Post hoc Newman–Keuls analyses confirmed significant pairwise differences predominantly between MHNW and MUO, MHNW and MHO, as well as NWO and MUO, further supporting the heterogeneity of body composition profiles despite similar BMI values in certain groups.

**Figure 1:**
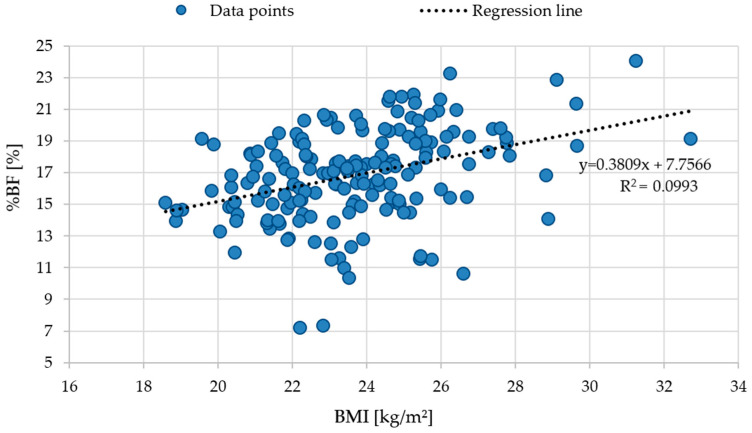
Relationship between body mass index (BMI) and percentage of body fat (%BF) in the total study cohort. Abbreviations: BMI—Body Mass Index (kg/m^2^); %BF—percent body fat.

**Figure 2:**
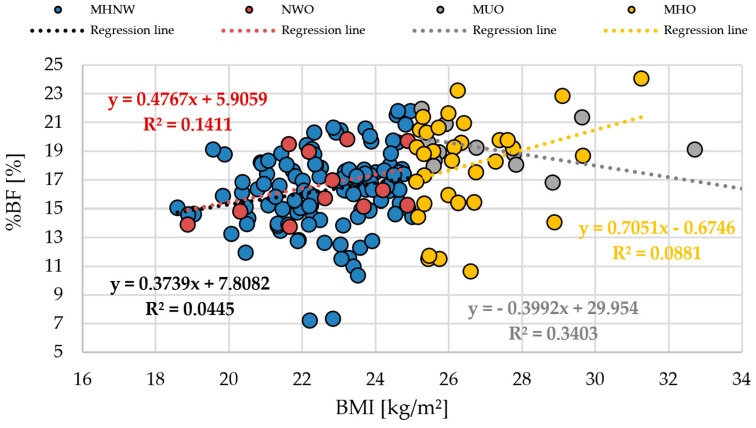
Relationship between BMI and %BF stratified by obesity phenotypes. Abbreviations: BMI—Body Mass Index (kg/m^2^); %BF—percent body fat; MHNW—normal weight with favorable anthropometric profile; NWO—normal weight obesity phenotype; MHO—obesity with favorable anthropometric profile; MUO—obesity with unfavorable anthropometric profile.

To further examine the relationship between body mass index (BMI) and body fat percentage (%BF), scatterplots were generated for both the entire cohort and the individual phenotypic subgroups (Figure 1 and Figure 2). These visualizations allow for the assessment of interindividual variability in adiposity relative to BMI and illustrate how this association differs across phenotypes.

Figure 1 depicts the relationship in the total sample (n = 174), showing a moderately positive correlation between BMI and %BF (y = 0.3809x + 7.7566, R^2^ = 0.0993), although substantial variability in %BF was observed at any given BMI. Figure 2 presents the BMI–%BF associations stratified by obesity phenotypes. In the normal weight group with a favorable anthropometric profile (MHNW), a weak positive relationship was observed (y = 0.3739x + 7.8082, R^2^ = 0.0445). The normal weight obesity (NWO) subgroup demonstrated a somewhat stronger positive association (y = 0.4767x + 5.9059, R^2^ = 0.1411). In contrast, individuals with obesity and an unfavorable anthropometric profile (MUO) displayed a negative regression slope (y = −0.3992x + 29.954, R^2^ = 0.3403), whereas the obesity group with a favorable anthropometric profile (MHO) showed a positive association (y = 0.7051x − 0.6746, R^2^ = 0.0881).

These findings indicate that BMI accounts for only a limited proportion of the variability in %BF, particularly among the MUO and MHNW subgroups. In NWO and MHO, the BMI–%BF relationship was stronger, although still moderate. In MUO, however, the negative slope combined with a higher R^2^ value suggests a distinct pattern in which higher BMI does not necessarily correspond to higher adiposity. Across all groups, differences in slope and explained variance demonstrate that individuals with similar BMI may present markedly different levels of %BF, underscoring the heterogeneity of body composition profiles within the cohort.

**Figure 3:**
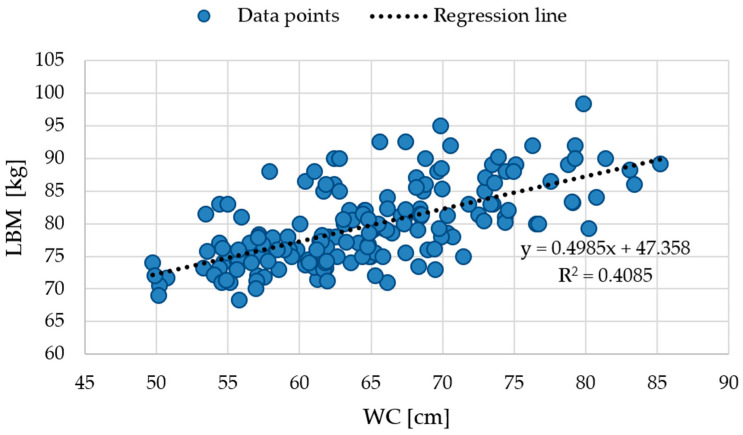
Scatter plot illustrating the relationship between waist circumference and lean body mass in the total cohort. Abbreviations: WC—waist circumference (cm); LBM—lean body mass (kg).

**Figure 4:**
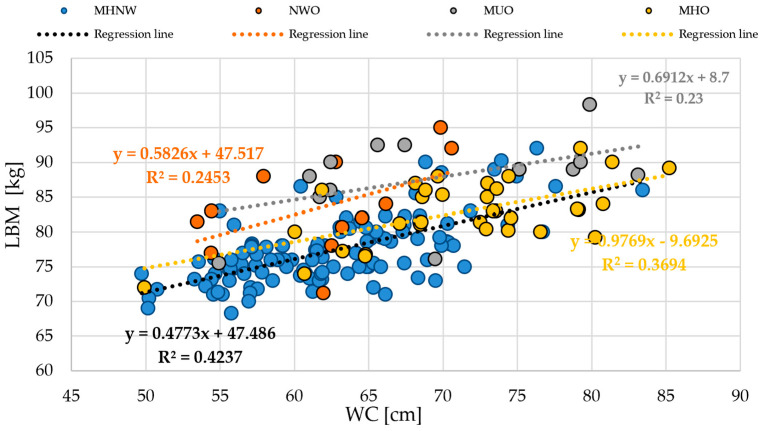
Scatter plot showing the relationship between waist circumference and lean body mass stratified by anthropometric phenotypes. Abbreviations: WC—waist circumference (cm); LBM—lean body mass (kg); MHNW—normal weight with favorable anthropometric profile; NWO—normal weight obesity phenotype; MHO—obesity with favorable anthropometric profile; MUO—obesity with unfavorable anthropometric profile.

To investigate the relationship between lean body mass (LBM, kg) and waist circumference (WC, cm), scatterplots were generated for both the entire cohort and the individual anthropometric phenotypes (Figure 3 and Figure 4). These visualizations allow assessment of how lean mass changes with increasing central adiposity and facilitate comparison between phenotypic groups.

Figure 3 illustrates the LBM–WC relationship in the total sample (n = 174), showing a positive correlation described by the regression equation y = 0.4985x + 47.358 (R^2^ = 0.4085), indicating that waist circumference explains a moderate proportion of the variability in lean body mass.

Figure 4 presents the same relationship stratified by anthropometric phenotypes. In the MHNW (normal weight with a favorable anthropometric profile) group, the regression equation was y = 0.4773x + 47.486 (R^2^ = 0.4237). In the NWO (normal weight obesity phenotype) subgroup, y = 0.5826x + 47.517 (R^2^ = 0.2453), whereas in the MUO (obesity with an unfavorable anthropometric profile) group, y = 0.6912x + 8.7 (R^2^ = 0.23). In the MHO (obesity with a favorable anthropometric profile) subgroup, the relationship was described by y = 0.9769x − 9.6925 (R^2^ = 0.3694).

These findings indicate that the proportion of LBM variability explained by WC differs across phenotypes. MHNW and MHO participants exhibited relatively higher R^2^ values, reflecting more consistent relationships between waist circumference and lean mass, whereas the NWO and MUO subgroups demonstrated lower R^2^ values, indicating greater variability in LBM relative to WC within these phenotypes.

To further explore the independent contributions of anthropometric indices, a multiple linear regression model was conducted (Table 3). The regression model was statistically significant (F(4173) = 27.47, *p* < 0.0001), explaining 38.8% of the variance in body fat percentage. WC was the strongest independent predictor of %BF (β = 0.345, *p* < 0.001), followed by WHR (β = −9.84, *p* = 0.025). In contrast, BMI (β = −0.23, *p* = 0.118) and WHtR (β = 8.47, *p* = 0.556) were not significant after adjustment for other indices. These results indicate that central adiposity measures provide unique information about body fatness beyond BMI.

## 4. Discussion

The findings of this study clearly indicate that the traditional BMI is a limited tool for assessing body composition–related risk in young, physically active adults. Despite a normal mean BMI, participants exhibited considerable heterogeneity in body composition, including body fat percentage (%BF) and lean body mass (LBM), highlighting the presence of NWO and MUO phenotypes even within an active population—a phenomenon rarely reported in the literature. Regression analyses between BMI and %BF revealed low coefficients of determination (R^2^), particularly in the MUO and MHNW groups, indicating that BMI does not fully capture individual differences in fat content and distribution [21,22]. The results of the multiple regression analysis further confirmed these findings by demonstrating that waist circumference (WC) and waist-to-hip ratio (WHR), rather than BMI, were the strongest independent predictors of body fat percentage. In contrast, BMI and WHtR did not significantly contribute to the prediction of %BF after adjustment for other indices. This highlights the limited diagnostic value of BMI as a standalone measure and underscores the importance of incorporating central adiposity indicators when assessing metabolic risk in young adults.

These observations are consistent with existing literature on anthropometric phenotypes. The NWO phenotype, characterized by normal BMI but elevated %BF, was present in 6.9% of participants, indicating unfavorable body composition despite normal weight in young, active adults. Similar findings were reported by Hadaye et al. [23], who demonstrated that NWO in this age group is associated with increased cardiometabolic risk. Kaczmarek [24] emphasized the role of lifestyle and dietary habits in NWO development, while Aruna et al. [25] linked this phenotype to adverse changes in cardiovascular parameters among young adults. The MUO phenotype, encompassing participants with elevated BMI and unfavorable anthropometric profiles, occurred in 8.0% of the sample, further underscoring the variability in anthropometric health profiles within this population. Systematic reviews and meta-analyses [26] confirm that MUO individuals have substantially higher cardiometabolic risk compared with those with normal BMI and favorable body composition, justifying the inclusion of body composition parameters in assessments related to cardiometabolic health.

Analysis of lean body mass provides critical insights into protective mechanisms related to body composition and cardiometabolic function. Higher LBM, particularly observed in MHO and MHNW groups, was associated with more favorable fat distribution indices (WHR, WHtR), may reflect a more advantageous anthropometric profile. Lean mass, particularly skeletal muscle, plays a central role in glucose homeostasis, lipid metabolism, and inflammation regulation [27,28,29]. Individuals with well-developed muscle mass may exhibit an MHO phenotype, characterized by elevated BMI but maintained favorable anthropometric features, consistent with our findings.

From a clinical and preventive perspective, the data underscore the need for multiparametric assessment of body composition and fat distribution, especially in young, active adults such as physical education students—a group rarely studied in international research. Numerous studies have emphasized that BMI-based classification alone has limited diagnostic value, failing to identify individuals with NWO or MUO phenotypes, who, despite normal body weight, may be at increased cardiometabolic risk according to previous research [26,30,31,32,33]. Incorporating body composition and fat distribution measurements, using methods such as bioelectrical impedance analysis (BIA), dual-energy X-ray absorptiometry (DEXA), or anthropometric waist measurements, enhances diagnostic sensitivity and more anthropometric risk profiles [16,34]. The ratio of fat-free to fat mass (FFMI/FMI) also remains an important indicator for assessing variation in body composition relevant to cardiometabolic health, as supported by recent analyses [35,36].

These findings also have practical implications for exercise programming and intervention planning in young, active populations. Evidence shows that increasing muscle mass and maintaining appropriate fat distribution improve insulin sensitivity, reduce blood pressure, and decrease inflammatory markers [37,38]. Studies in Poland corroborate these associations—Jaremków et al. [39] demonstrated that physical activity levels among medical students significantly influenced body composition parameters such as muscle mass, lean body mass, and fat percentage, highlighting the need for personalized approaches even in young, seemingly healthy populations. Given the observed heterogeneity of anthropometric phenotypes, individualized training and dietary programs are particularly important, even in individuals who appear healthy and whose BMI does not accurately reflect unfavorable body composition [40]. From a practical perspective, these results emphasize the importance of including routine assessment of body composition and fat distribution in academic physical education programs and preventive health evaluations. Implementing targeted exercise interventions focused on strength and neuromuscular training may help optimize muscle-to-fat ratios and support cardiometabolic function in young adults. In addition, integrating educational components that promote balanced nutrition and awareness of factors influencing cardiometabolic health could further enhance the effectiveness of such programs. While these findings provide valuable insights into the anthropometric variability of physically active young men, certain methodological constraints should be considered when interpreting the results. It is important to acknowledge the limitations of this study. The analysis included only young men, which limits the generalizability of the findings to women and other age groups. This design choice resulted from the demographic structure of the study population—at the Faculty of Physical Education, male students constitute the vast majority, while female students represent only a small proportion. Including women in the present analysis would have produced very small female subgroups, thereby reducing statistical power and compromising the reliability of intergroup comparisons. To ensure methodological consistency and robust statistical interpretation, the current study focused on a homogeneous male sample. Nevertheless, future research should involve larger, multicenter, and mixed-gender cohorts to verify whether similar anthropometric phenotypes are observed in women and to explore potential sex-specific differences in body composition and cardiometabolic risk. It should also be noted that the relatively small sizes of the NWO and MUO subgroups may have limited statistical power. Therefore, the corresponding results should be interpreted with caution and verified in larger, multicenter samples. Additionally, the lack of biochemical measurements, such as glucose, insulin, or lipid profiles, precludes a comprehensive assessment of metabolic status. Another limitation concerns the absence of detailed data on exercise frequency, intensity, and type. However, all participants were students of the Faculty of Physical Education, where regular physical activity and sports training are integral parts of the curriculum. Students of this program typically engage in approximately 10 h of structured physical activity per week, which substantially exceeds the mandatory 2 h of physical education required for students of non-sport-related faculties. Therefore, the analyzed group can be considered physically active and relatively homogeneous in terms of habitual exercise engagement. Future studies should include standardized assessments of physical activity to better capture its influence on body composition and phenotype classification. Future studies should also incorporate biochemical parameters, as well as detailed analyses of physical activity levels, training types, and genetic and environmental factors influencing anthropometric phenotypes. It should also be noted that bioelectrical impedance analysis (BIA), although practical and noninvasive, is not a gold-standard method for assessing body composition compared with dual-energy X-ray absorptiometry (DXA) or magnetic resonance imaging (MRI). Moreover, raw impedance parameters (resistance, reactance, and phase angle) were not collected or analyzed in the present study, which limits the possibility of further validation and detailed analysis of body composition components.

In conclusion, the results of this study underscore the limitations of BMI as a sole indicator of body composition and fat distribution and highlight the heterogeneity of obesity-related anthropometric phenotypes in young, active adults. Central adiposity indices, particularly WC and WHR, emerged as the strongest independent predictors of body fat percentage, outperforming BMI. Lean body mass and regional fat distribution represent key factors differentiating body composition profiles and should be incorporated into comprehensive health assessments. These findings carry both clinical and preventive significance, emphasizing the necessity of a multiparametric diagnostic approach, monitoring of body composition, and individualized exercise and dietary interventions in young adult populations.

## 5. Conclusions

Based on the findings of this study, it is evident that the traditional BMI is insufficient as a standalone indicator for assessing body composition and fat distribution in young, physically active adults. The observed heterogeneity of anthropometric phenotypes, including both NWO and MUO, indicates that individuals with normal body weight may still present unfavorable body composition characteristics. Importantly, central adiposity indicators—particularly waist circumference and waist-to-hip ratio—were identified as the strongest independent predictors of body fat percentage, further underscoring the limited diagnostic value of BMI alone. Lean body mass and regional fat distribution play a key role in differentiating body composition profiles, and their inclusion in routine anthropometric assessments allows for more precise identification of individuals whose body composition may predispose them to adverse health outcomes. The results also underscore the importance of physical activity and the individualization of training programs and dietary interventions, even in seemingly healthy populations, highlighting important implications for preventive health practice. Future research should extend the analysis to women and broader age groups and incorporate biochemical measurements to enable a more comprehensive assessment of metabolic status and better understand the mechanisms shaping anthropometric phenotypes in young adult populations.

## Figures and Tables

**Table 1:** General characteristics and body composition of the study participants (n = 174 men).

	Mean	SD	Min	Max
Age	20.88	0.86	19.70	23.59
BH	181.15	7.26	164.50	199.50
BW	78.17	10.46	57.70	119.40
BMI	23.79	2.58	18.59	35.27
WC	79.74	6.16	68.30	99.00
HC	97.57	6.95	78.50	118.00
WHR	0.82	0.07	0.73	1.10
WHtR	0.44	0.03	0.38	0.54
%BF	16.77	2.94	7.23	24.07
%FFM	83.21	2.94	75.93	92.77
FM	13.22	3.39	4.83	24.12
FMI	4.01	0.94	1.60	7.52
LBM	64.95	8.20	49.72	103.10
FFMI	19.78	2.08	15.78	30.45

Abbreviations: Age—age (years); BH—body height (cm); BW—body weight (kg); BMI—Body Mass Index (kg/m^2^); WC—waist circumference (cm); HC—hip circumference (cm); WHR—Waist-to-Hip Ratio; WHtR—Waist-to-Height Ratio; %BF—percent body fat; %FFM—percent fat-free mass; FM—fat mass (kg); FMI—Fat Mass Index (kg/m^2^); LBM—lean body mass (kg); FFMI—Fat-Free Mass Index (kg/m^2^).

**Table 2:** Distribution of participants across obesity-related anthropometric phenotypes and comparative analysis of anthropometric and body composition parameters.

	MHNW (n 116)		NWO (n 12)		MUO (n 14)		MHO (n 33)	
	mean	SD	mean	SD	mean	SD	mean	SD
Age	20.84	0.85	20.61	0.68	21.42	1.13	20.87	0.78
BH	180.83	7.35	181.46	6.83	178.44	7.22	183.32	6.81
BW	74.12	7.81	74.34	7.31	89.48	11.87	89.02	7.58
BMI	22.63	1.51	22.58	1.80	28.07	3.07	26.47	1.46
WC	76.78	4.33	84.33	5.67	90.48	4.02	83.92	3.63
HC	96.63	4.59	85.18	5.10	98.94	10.21	104.79	4.50
WHR	0.80	0.04	0.99	0.06	0.92	0.08	0.80	0.04
WHtR	0.42	0.02	0.46	0.02	0.51	0.02	0.46	0.02
%BF	16.27	2.68	16.67	2.28	18.17	3.18	17.99	3.48
%FFM	83.73	2.68	83.33	2.28	81.83	3.18	82.01	3.48
FM	12.13	2.74	12.44	2.37	16.11	2.74	16.09	3.74
FMI	3.69	0.71	3.78	0.67	5.05	0.80	4.78	1.05
LBM	61.98	6.06	61.91	5.77	73.37	11.47	72.94	6.03
FFMI	18.94	1.28	18.80	1.41	23.02	3.16	21.69	1.26
***One-way analysis of variance (ANOVA) and Newman–Keuls post hoc test.***								
		Anova	I–II	I–III	I–IV	II–III	II–IV	III–IV
Age		2.36	1.26	3.40 *	1.25	3.42 *	1.28	2.86
BH		1.74	0.41	1.66	2.48	1.51	1.08	3.00
BW		39.21	0.13	9.46 *	13.15 *	6.70 *	7.59 *	0.25
BMI		77.83	0.14	16.09 *	16.28 *	11.68 *	9.65 *	4.20 *
WC		62.79	8.21 *	15.98 *	11.94 *	5.16 *	0.40	6.79 *
HC		44.45	10.17 *	2.20	11.14 *	9.42 *	15.67 *	4.94 *
WHR		86.81	19.37 *	13.11 *	-	5.50 *	17.42 *	11.67
WHtR		109.91	9.33 *	22.49 *	14.34 *	8.99 *	-	11.08 *
%BF		4.30	0.65	3.32	4.31 *	1.88	1.93	0.28
%FFM		4.30	0.65	3.32	4.31 *	1.88	1.93	0.28
FM		388.26	20.16 *	19.65 *	40.65 *	5.78 *	5.32 *	1.52
FMI		20.70	0.49	6.78 *	9.68 *	4.50 *	5.22 *	0.03
LBM		32.33	0.05	8.62 *	11.89 *	6.24 *	7.00 *	0.29
FFMI		52.60	0.43	13.49 *	13.04 *	10.03 *	8.02 *	3.90 *

Abbreviations: MHNW—normal weight with favorable anthropometric profile; NWO—normal weight obesity phenotype; MHO—obesity with favorable anthropometric profile; MUO—obesity with unfavorable anthropometric profile; Age—age (years); BH—body height (cm); BW—body weight (kg); BMI—Body Mass Index (kg/m^2^); WC—waist circumference (cm); HC—hip circumference (cm); WHR—waist-to-hip ratio; WHtR—waist-to-height ratio; %BF—percent body fat; %FFM—percent fat-free mass; FM—fat mass (kg); FMI—Fat Mass Index (kg/m^2^); LBM—lean body mass (kg); FFMI—Fat-Free Mass Index (kg/m^2^); * statistically significant difference at *p* < 0.05.

**Table 3:** Multivariate linear regression predicting body fat percentage (%BF).

Variable	Coefficient (β)	95% CI	*p*-Value
Intercept	−0.88	−4.67 to 2.91	0.647
BMI	−0.23	−0.52 to 0.06	0.118
WC	0.35	0.21 to 0.48	<0.001
WHR	−9.84	−18.40 to −1.28	0.025
WHtR	8.47	−19.88 to 36.82	0.556

Model summary: R^2^ = 0.388, Adjusted R^2^ = 0.374; F(4173) = 27.47, *p* < 0.0001. Abbreviations: %BF—body fat percentage; BMI—body mass index; WC—waist circumference; WHR—waist-to-hip ratio; WHtR—waist-to-height ratio.

## Data Availability

Dataset is available on request due to restrictions.
